# Breast Cancer Information Grand Round for Survivorship (BIG-S) prospective cohort

**DOI:** 10.1007/s10654-025-01262-5

**Published:** 2025-07-08

**Authors:** Danbee Kang, Juwon Park, Hyunsoo Kim, Jeong Eon Lee, Seok Jin Nam, Seok Won Kim, Jonghan Yu, Byung Joo Chae, Jai Min Ryu, Juhee Cho, Se Kyung Lee

**Affiliations:** 1 Center for Clinical Epidemiology, Samsung Medical Center, Sungkyunkwan University School of Medicine, Seoul, Republic of Korea; 2 Department of Clinical Research Design & Evaluation, SAIHST, Sungkyunkwan University, Seoul, Republic of Korea; 3 Trend Sensing and Risk Modeling Center, Institution of Quality of Life in Cancer, Samsung Medical Center, Sungkyunkwan University School of Medicine, Seoul, Republic of Korea; 4 Department of Surgery, Samsung Medical Center, Sungkyunkwan University School of Medicine, Seoul, Republic of Korea; 5 Division of Breast Surgery, Department of Surgery, Samsung Medical Center, Sungkyunkwan University School of Medicine, Seoul, Republic of Korea

**Keywords:** Breast cancer, Survivorship, Quality of life, Prospective cohort

## Abstract

Existing evidence indicates that long-term effects of breast cancer treatment can significantly impact survivors’ ability to fulfill their personal, familial, and social roles. However, few studies comprehensively integrate patient-reported outcomes (PROs) and real-world healthcare utilization data, particularly in Asian populations. Thus, we established a prospective cohort, the Breast Cancer Information Grand Round for Survivorship (BIG-S), to address these gaps and introduce this resource. The prospective BIG-S cohort recruited patients newly diagnosed with breast cancer at Samsung Medical Center starting in November 2018. Clinical data, recurrence, and healthcare utilization were systematically collected from electronic medical records by trained researchers, and body composition was measured using multifrequency bioelectrical impedance analysis. PROs were assessed following recommendations from the International Consortium for Health Outcomes Measurement (ICHOM). These included health-related quality of life, physical, psychological, social, and cognitive functions, symptoms, healthy behaviors, financial difficulty, spiritual well-being, and cancer adaptation, using validated questionnaires. A total of 2,749 patients participated, with an average age of 49.7 years. The mean quality-of-life score at diagnosis was 55.6, indicating moderate general well-being, and improved gradually to 68.2 at four years post-diagnosis. At baseline, participants showed high physical, cognitive, and role functioning scores but had relatively lower emotional and social functioning scores. Over four years, emotional and social functioning improved, whereas cognitive and role functioning declined. Survivors initially reported low sexual functioning, sexual enjoyment, and future perspectives, all of which significantly decreased during follow-up. Fatigue and insomnia were persistent throughout the observation period. Healthcare utilization initially concentrated on plastic surgery and rehabilitation medicine and increasingly shifted towards gynecology, family medicine, and psychiatry after two years. The BIG-S cohort uniquely integrates clinical data, PROs, and healthcare utilization patterns, offering comprehensive insights into breast cancer survivorship trajectories. Findings from BIG-S are expected to guide targeted interventions and inform tailored survivorship care strategies, especially for Asian breast cancer survivors.

## Background

Breast cancer is the most commonly diagnosed cancer in females aged < 40 years [1]. For many survivors, especially younger females, returning to normal life includes personal, family, and social roles and work responsibilities [2]. Existing evidence on the long-term effects of treatment, including fatigue, cognitive impairment, psychological distress, and physical limitations, can affect a survivor’s ability to engage in their roles as mothers, wives, and caregivers [3, 4].

However, studies lack detailed clinical information and do not fully capture the management of various toxicities and the long-term effects of treatment. According to an American Society of Clinical Oncology survey [5], little evidence exists on the patterns and quality of survivorship care [6]. The consensus of the National Cancer Institute reported a need for research to identify the social functioning needs of long-term cancer survivors and to implement and integrate psychosocial interventions in real-world settings [7].

Among the available registries, the Kaiser Permanente Breast Cancer Survivors Cohort could link data from electronic medical records, cancer registries, and the National Death Index; however, there was a lack of patient-reported outcomes. Although Sporadic and Hereditary Breast Cancer [8] and Harvard [9] have collected extensive data on tumor characteristics and treatment, they may have limited information on other potentially relevant factors, such as lifestyle or environmental exposures. In contrast, recently, patient-centered cohorts like CANcer TOxicities have provided valuable insights about treatment-related toxicities and long-term health outcomes; [10] however, they lack the comprehensive integration of real-world healthcare utilization data. Moreover, a lack of an Asian breast cancer registry comparable to that available for Western patients exists, although approximately half of Asian patients with breast cancer are premenopausal, in contrast to only 15–30% of Western patients with breast cancer [11]. Furthermore, differences have been observed in tumor immune microenvironments between Asian and Western populations [12].

Recognizing the distinct clinical challenges and research gaps in breast cancer, we established the Breast Cancer Information Grand Round for Survivorship (BIG-S) registry. This registry collected prospective data from 3,894 patients who had been followed up for a minimum of 10 years. The BIG-S uniquely integrates detailed clinical data, patient-reported outcomes, and real-world healthcare utilization patterns.

## Materials and methods

### Study design and participants

This prospective cohort included patients newly diagnosed with histologically confirmed breast cancer without prior treatment at Samsung Medical Center (SMC) in November 2018. The BIG-S study included survivors aged > 20 years without secondary cancer, metastasis, or recurrence.

The study was approved by the Institutional Review Board of the SMC (SMC-2019-08-009-020). We strictly adhered to the ethical guidelines outlined in the latest version of the Declaration of Helsinki and the International Conference on Harmonization Guidelines for Good Clinical Practice.

### Measurement

#### Clinical information

Clinical information, including pregnancy, birth history, body mass index, year of diagnosis, Eastern Cooperative Oncology Group Performance Status score, comorbidities, pathological stage based on the American Joint Committee on Cancer staging manual, subtype, breast cancer susceptibility gene 1 and/or 2, and treatment modalities, was regularly updated by a trained data manager.

In terms of pathologic data, two experienced pathologists reviewed and determined the primary tumor characteristics based on size, axillary nodal status, and receptor status including estrogen receptor (ER), progesterone receptor (PR), and human epidermal growth factor receptor 2 (HER2) using immunohistochemical (IHC) staining. ER positivity (ER+) and PR positivity (PR+) were defined as Allred scores of 3–8 based on IHC staining with antibodies against ER (Immunotech, Marseille, France) and PR (Novocastra, Newcastle upon Tyne, UK), respectively. HER2 status was evaluated using appropriate antibodies (Dako, Carpinteria, CA, USA) and/or silver in situ hybridization (SISH). Additionally, HER2 grades 0 and 1+ indicate negative results, whereas grade 3+ indicates positive results. Grade 2+ was equivocal and further confirmed using FISH. SISH was used to confirm HER2 amplification in patients with 2+ staining. Triple-negative breast cancer was defined as breast cancer with negative ER (ER−), PR (PR−), and HER2 expression (Table 1). Pathological data, including IHC results, were collected at the time of recurrence or distant metastasis.

**Table 1:** Overview of collected variable

Domains	Contents	Measurement Methods
Recruited information	Date at enrollment	Survey
Clinical Characteristics		EMR
Clinic Visits	Cardiology, Dentistry, Dermatology, Endocrinology and Metabolism, Family Medicine, Gastroenterology, Gynecology & Obstetrics, Health Promotion Center, Infectious Diseases, Laboratory Medicine and Genetics, Neurology, Neurosurgery, Ophthalmology, Orthopedic Surgery, Otorhinolaryngology, Plastic Surgery, Psychiatry, Pulmonary and Critical Care Medicine, Radiology, Rehabilitation Medicine, Thoracic Surgery	EMR
Socio-Demographic	Sex, birthdate, age at diagnosis, weight, height, body mass index	Survey
	Marital status, education, employment status, income, private insurance status	
Comorbidities	Diabetes mellitus, Hypertension, Hyperlipidemia, Cardiovascular disease, Liver disease, Pulmonary disease, Gastrointestinal disease, Cerebrovascular disease, Chronic renal disease, Musculoskeletal disease, Thyroid disease, Eye disease, Others	Survey
Health Behavior	Smoking status, Drinking status	Survey
Female Health	Menopausal status, history of birth, number of birth	Survey
Cognitive Impairment: Subjective Memory Complaints	Functional Assessment of Cancer Therapy (FACT)	Survey
Nutrition and Eating Behavior	Healthy Eating Index (HEI)	Survey
Physical Activity	International Physical Activity Questionnaire (IPAQ)	Survey
Quality of life	EORTC QLQ C30	Survey
Quality of Life in Patients with Breast Cancer	EORTC QLQ BR23	Survey
Psychological and Spiritual Problems	Quality of Life Cancer Survivorship - Spiritual Well-Being (QLQ CS-SWB)	Survey
	Fear of Cancer Recurrence Inventory-Short Form (FCRI-SF)	
	Patient Health Questionnaire 9 (PHQ9)	
	Mini-Mental Adjustment to Cancer Scale (Mini-MAC)	
Disclose	Talk to parents, partner, child, brother, in-law, friends, neighbor, boss, colleague, customer or none	Survey
Financial distress	Comprehensive Score for Financial Toxicity (FACIT-COST)	Survey

EMR: electronic medical records

#### Body composition

At each visits, we used bioelectrical impedance analysis to determine body composition using a multifrequency bioelectrical impedance device according to the manufacturer’s instructions (InBody 720; InBody Co., Ltd., Seoul, Korea).

#### Health-related quality of life, physical, psychological, social, and cognitive function, as well as symptoms

In patient-reported outcome, we followed by recommendation from International Consortium for Health Outcomes Measurement (ICHOM) [13]. To collect the Patient Reported Outcome (PRO), we used a digital platform with the mobile phones of the patients.

The European Organisation for Research and Treatment of Cancer (EORTC) Quality of Life Questionnaire-Core 30 (QLQ-C30) was used to measure physical, emotional, cognitive, and social functioning; ability to work; and overall well-being [14]. Breast cancer-specific functions such as body image, sexual functioning, future perspective, systemic therapy side effects, arm and breast symptoms, and upset by hair loss were measured using the Breast Cancer-Specific Module (BR23) [14]. We used FACT-Cognitive to estimate cognitive function, which was constructed of 37 questions. The sub-items included 20 questions on perceived cognitive impairment, 9 on perceived cognitive function, 4 on other people’s views, and 4 on the impact on quality of life. The tool uses a 5-point scale ranging from 0 (never or not at all) to 4 (many times a day or very much so),” with the total score from each subscale summed to represent the overall cognitive function, with higher scores indicating better cognitive function.

The EORTC QLQ C30 includes cancer-related symptoms such as fatigue, nausea and vomiting, pain, dyspnea, insomnia, appetite loss, constipation, diarrhea, and financial difficulties. Breast cancer-specific symptoms such as side effects of systemic therapy, arm and breast symptoms, and upset by hair loss were measured using the Breast Cancer-Specific Module (BR23).

The Menopause Rating Scale (MRS) was used to measure menopausal symptoms. The MRS includes 11 items in three dimensions: somatic-vegetative, psychological, and urogenital. Composite scores (range: 0–44) were calculated by adding the scores of the items from the respective dimensions.

#### Healthy behavior

We used the Centers for Disease Control and Prevention and developed an 11-question dietary checklist using the Healthy Eating Index, based on the 10 items of the Healthy Eating Index.

Physical activity was measured using the Physical Activity International Physical Activity Questionnaire developed by the International Consensus Organization to comprehensively and objectively assess health-related physical activity and usual physical activity—the questionnaire was surveyed using the Short Last 7 Days Self-Administered Form.

#### Financial difficulty

We used the Functional Assessment of Chronic Illness Therapy (FACIT) -COmprehensive Score for Financial Toxicity (COST) to assess financial toxicity, a 12-item questionnaire [15]. The COST-K is a valid and reliable instrument to measure financial toxicity in disease-free breast cancer survivors [16].

Insurance status was defined based on whether the individuals had private insurance. Private insurance status.

#### Spiritual well-being and adaptation to cancer

Spiritual well-being was evaluated using three questions from the Spiritual Well-Being Domain of the Korean version of the QoL of Cancer Survivors questionnaire [17]. From these, we selected four questions related to spiritual health. The Mini-Mental Adjustment to Cancer Scale (Mini-MAC) was used to capture how a person responded to a cancer diagnosis. It consists of 29 items on five scales: helpless/hopeless, anxious preoccupation, fighting spirit, cognitive avoidance, and fatalism.

The severity of the fear of cancer recurrence was assessed using the 9-item Korean version of the Fear of Cancer Recurrence Inventory-Short Form. Each item is rated on a 5-point scale ranging from 0 (“not at all” to 4 “very much ”) [18].

Depression was assessed using the Patient Health Questionnaire 9 (PHQ9), a standardized instrument for measuring depressive symptoms. Higher PHQ9 scores reflect greater severity of depression, with a score of 5 or above (score range: 0–27) indicating the presence of depressive symptoms [19].

#### Health care utilization

Healthcare utilization information was identified using the electronic medical records (EMR) of all patients. We have plant-to-link cohort data from Korea’s National Health Insurance and Health Insurance Review and Assessment Service databases. These provide a unique opportunity to examine healthcare utilization, long-term outcomes, and broader societal impacts, offering insights that are often absent in other studies due to fragmented healthcare systems.

#### Clinical outcomes

Survival and recurrence information were updated regularly by trained data managers using EMR for all patients. For recurrence monitoring, tumor marker tests and imaging such as CT and bone scan were performed every 6–12 months. If necessary, a PET-CT scan was performed to determine recurrence. If suspicious findings of recurrence were observed on imaging, a biopsy was performed for diagnostic confirmation. The recurrence date was defined as the day when clinicians confirmed recurrence according to the imaging or pathological results and is described in the chart. We also used official death certificates obtained from the Ministry of Strategy and Finance of South Korea to confirm the vital status.

Disease-free survival was defined as the period from the date of treatment initiation until recurrence or death, whichever occurred first. Patients who survived without recurrence at the time of the final analysis were evaluated at the last tumor assessment date. Overall survival was defined as the period from the start of treatment to the date of death. Patients who survived at the time of the final analysis were assessed at the time of their last survival.

### Statistical analyses

Cohort data are represented using descriptive analysis, displaying the mean (standard deviation) and number (percentage) of continuous and categorical variables. By combining the questionnaires and EMR information of patients with breast cancer who participated in the study, we will identify the survival period of the target group (survivors immediately after the end of treatment, 6 months, and 1, 2, 3, and 4 years after the end of treatment) and perform descriptive analyses such as frequency analysis and mean analysis by the target group.

Statistical significance was set at *p* <.05, and two-tailed tests were used for all calculations. Statistical analyses were performed using R 4.1.2 (R Foundation for Statistical Computing, Vienna, Austria).

## Results

### Study population

A total of 2,749 patients met the eligibility criteria and participated in the study. The average (standard deviation) age was 49.65 (8.58) years. Most participants were married (66.1%), with similar proportions across the neoadjuvant and adjuvant cohorts (Table 2).

**Table 2:** Characteristics of study participants, N (%)

Characteristics	Overall
	(*N* = 2,749)
Age, mean (SD)	49.65 (8.58)
Body Mass Index (kg/m^2^)	
< 18.5	111 (4.0)
18.5–23	1376 (50.1)
≥ 23	1261 (45.9)
Missing	1 (0.0)
Marital status at diagnosis	
Single	208 (7.6)
Married	1828 (66.5)
Divorced	143 (5.2)
Bereavement	66 (2.4)
Missing	504 (18.3)
Education, ≥university	1320 (48.0)
Household monthly income ($)	
Less than 1,000	205 (7.5)
1,000–2,000	330 (12.0)
2,000–4,000	702 (25.5)
4,000–6,000	515 (18.7)
6,000 and above	489 (17.8)
Missing	508 (18.5)
Job-status at diagnosis	
House-wife	830 (30.2)
Employed	810 (29.5)
Sick leaves	170 (6.2)
Self-employed	303 (11.0)
Retired	96 (3.5)
Others	36 (1.3)
Missing	504 (18.3)
Private insurance status	
Yes	2117 (77.0)
No	127 (4.6)
Missing	505 (18.4)
Smoking status, ever	219 (8.0)
Drinking status, current drinker	928 (33.8)
Physical Activities	
Inactive	761 (27.7)
Minimally active	767 (27.9)
Active	337 (12.3)
Missing	884 (32.2)
Comorbidity	
Diabetes mellitus	100 (3.6)
Hypertension	259 (9.4)
Hyperlipidemia	270 (9.8)
Cardiovascular disease	19 (0.7)
Liver disease	39 (1.4)
Pulmonary disease	22 (0.8)
Gastrointestinal disease	143 (5.2)
Cerebrovascular disease	9 (0.3)
Chronic renal disease	9 (0.3)
Thyroid disease	133 (4.8)
Eye disease	38 (1.4)
Others	118 (4.3)
Menopausal status	
Premenopausal	1582 (57.5)
Postmenopausal	1161 (42.2)
Missing	6 (0.2)
Stage	
non-NAC	
0	318 (11.6)
I	1033 (37.6)
II	607 (22.1)
III	74 (2.7)
Unknown	7 (0.3)
NAC	
pCR	409 (14.9)
non-pCR	301 (10.9)
Type of surgery	
Total mastectomy	931 (33.9)
Reconstruction (*N* = 494)	494 (18.0)
Lumpectomy	1815 (66.0)
Others	3 (0.1)
Axilla surgery	
SLNB	2212 (80.5)
ALND	333 (12.1)
No operation	204 (7.4)
Treatment	
Chemotherapy	1321 (48.1)
Radiation therapy	2096 (76.2)
Hormone therapy	2308 (84.0)
Tamoxifen	1309 (47.6)
Aromatase Inhibitor	848 (30.8)
Gosereline	594 (21.6)
Targeted therapy	
Herceptin	386 (14.0)
Pertuzumab	315 (11.5)

Abbreviations: SLNB: sentinel lymph node biopsy; ALND: axillary lymph node dissection; NAC: neoadjuvant chemotherapy; PCR: Pathologic complete response

### PRO

The overall global health status/quality of life score at diagnosis was 55.62, indicating moderate general well-being among participants, which gradually improved over time, reaching 68.21 (Standard Deviation [SD] 18.74) at 4 years post-diagnosis (Table 3). At diagnosis, breast cancer survivors had high physical, cognitive, and role functioning scores, with averages of 84.96, 85.47, and 91.89, respectively. However, they had lower emotional and social functioning scores, with mean scores of 69.17 and 77.46, respectively. During follow-up, emotional and social functioning improved, whereas cognitive and role functioning declined. Breast cancer survivors had low sexual function, sexual enjoyment, and future perspectives at baseline, which significantly decreased during follow-up.

**Table 3:** Global health, functional, symptom, by time since diagnosis

Characteristics	Baseline	6 months	1 year	2 years	3 years	4 years
	(*N* = 2,749)	(*N* = 1,283)	(*N* = 2,449)	(*N* = 2,755)	(*N* = 1,975)	(*N* = 713)
Global health status/quality of life						
Global health status/quality of life	55.62 (21.53)	52.93 (20.82)	59.21 (19.71)	62.23 (19.79)	65.71 (20.45)	68.21 (18.74)
Functional scales						
Physical functioning	84.96 (11.97)	72.42 (18.26)	79.10 (14.14)	81.93 (13.11)	83.91 (12.32)	85.31 (11.59)
Cognitive functioning	85.47 (15.72)	78.11 (18.26)	78.25 (17.77)	78.31 (18.17)	78.71 (18.00)	79.73 (17.08)
Emotional functioning	69.26 (22.47)	72.01 (20.30)	74.47 (19.57)	75.50 (19.49)	76.88 (18.94)	77.98 (19.00)
Social functioning	77.56 (24.16)	68.61 (24.95)	75.59 (23.29)	78.45 (22.89)	81.72 (21.59)	83.90 (21.13)
Role functioning	91.89 (14.78)	74.51 (23.82)	80.16 (19.71)	82.83 (18.91)	85.35 (17.56)	86.43 (17.33)
Symptoms						
Fatigue	31.13 (20.69)	41.75 (22.50)	36.41 (20.15)	35.41 (19.93)	33.94 (19.81)	32.71 (18.44)
Nausea and vomiting	5.75 (10.61)	11.99 (15.03)	6.12 (10.60)	5.89 (10.73)	5.05 (9.48)	5.45 (9.90)
Pain	14.19 (16.80)	27.30 (21.81)	24.81 (19.77)	20.50 (18.56)	17.03 (16.41)	16.67 (15.83)
Dyspnea	12.92 (19.31)	24.11 (25.63)	15.68 (20.87)	14.06 (19.30)	13.28 (18.67)	13.92 (18.51)
Insomnia	28.56 (27.58)	41.06 (29.62)	38.64 (29.29)	35.95 (28.39)	34.94 (28.68)	34.05 (27.62)
Appetite loss	14.73 (20.37)	24.54 (27.97)	14.78 (21.04)	11.60 (19.10)	9.96 (17.75)	9.54 (17.18)
Constipation	15.34 (22.91)	18.76 (25.84)	16.14 (23.92)	17.58 (24.26)	17.87 (23.91)	18.04 (23.99)
Diarrhea	11.65 (19.20)	14.86 (22.91)	7.47 (16.00)	6.82 (15.06)	6.78 (14.55)	7.16 (14.54)
Financial problem	14.77 (23.72)	20.18 (26.50)	16.58 (23.93)	15.05 (22.94)	13.39 (22.00)	13.57 (22.78)
Breast cancer-specific functioning						
Body image	83.00 (19.81)	61.60 (28.40)	68.84 (26.34)	70.78 (25.54)	72.98 (24.35)	73.80 (24.55)
Sexual functioning	15.74 (19.94)	10.31 (16.82)	12.79 (18.54)	13.44 (19.42)	12.77 (18.83)	14.18 (20.92)
Sexual enjoyment	26.66 (26.44)	11.18 (19.76)	13.37 (21.42)	14.07 (21.73)	12.90 (20.64)	14.07 (22.48)
Future perspective	48.62 (30.30)	41.46 (28.52)	45.78 (28.66)	49.51 (27.81)	53.41 (27.12)	54.20 (26.36)
Breast cancer-specific symptoms						
Systemic therapy side effects	17.26 (13.75)	30.01 (19.09)	20.70 (14.45)	21.08 (14.31)	20.06 (14.37)	20.22 (14.13)
Upset by hair loss	25.03 (29.81)	50.10 (37.07)	38.58 (34.27)	41.31 (33.41)	42.88 (32.36)	42.95 (31.45)
Arm symptoms	18.77 (19.30)	30.69 (23.63)	31.94 (24.46)	26.63 (22.72)	22.25 (20.19)	20.79 (19.17)
Breast symptoms	14.40 (16.04)	17.37 (16.03)	21.45 (17.07)	17.10 (14.91)	14.23 (13.68)	12.72 (12.60)
Fear of recurrence	13.51 (7.22)	14.74 (6.81)	14.44 (6.81)	13.83 (6.80)	13.16 (6.79)	12.89 (6.52)
Depression	4.90 (5.00)	5.68 (4.93)	4.75 (4.56)	4.51 (4.49)	4.16 (4.22)	3.90 (4.11)
Menopause symptoms	8.49 (6.97)	12.15 (7.47)	12.09 (7.46)	12.02 (7.51)	11.53 (7.36)	10.98 (7.04)
Nutrition and eating behavior	28.34 (13.39)	25.37 (8.78)	25.82 (8.47)	26.58 (7.60)	27.15 (7.44)	27.79 (6.65)
Financial Toxicity	25.14 (7.62)	25.14 (8.10)	26.17 (7.67)	26.36 (7.83)	26.99 (7.72)	27.40 (7.81)
Cognitive impairment						
Perceived cognitive impairments	63.60 (9.77)	60.64 (11.23)	60.22 (11.36)	59.71 (12.21)	60.31 (11.27)	60.57 (11.20)
Impact on quality of life	10.94 (4.26)	11.51 (3.97)	11.96 (3.72)	12.12 (3.70)	12.43 (3.60)	12.49 (3.67)
Comments from others	15.49 (1.42)	15.44 (1.37)	15.40 (1.53)	15.33 (1.65)	15.44 (1.46)	15.54 (1.26)
Perceived cognitive abilities	18.19 (6.67)	17.09 (5.78)	16.86 (5.94)	16.85 (5.91)	17.35 (5.82)	17.47 (5.76)
Mental adjustment						
Helpless/Hopeless	12.57 (3.84)	12.89 (3.92)	12.55 (3.95)	12.41 (3.88)	12.11 (3.70)	11.95 (3.69)
Anxious Preoccupation	20.71 (4.98)	19.76 (4.65)	19.13 (4.61)	18.55 (4.69)	18.05 (4.62)	17.70 (4.64)
Fighting Spirit	11.74 (2.00)	11.77 (1.88)	11.70 (1.93)	11.67 (1.89)	11.67 (1.97)	11.71 (1.98)
Cognitive Avoidance	10.35 (2.43)	10.28 (2.42)	10.31 (2.38)	10.39 (2.44)	10.42 (2.50)	10.55 (2.45)
Fatalism	13.96 (2.58)	14.12 (2.48)	14.33 (2.50)	14.41 (2.47)	14.54 (2.58)	14.59 (2.56)

Values are expressed as mean (SD); the unique numbers were 992, 1966, 1805, 1414, and 560 at 6 months, 1 year, 2 years, 3 years, and 4 years after diagnosis, respectively

Regarding symptoms, fatigue and insomnia were prominent issues at diagnosis, with mean scores of 31.13 and 28.56, respectively. However, these symptoms did not resolve after 4 years. Breast cancer survivors consistently had systemic therapeutic side effects and arm and breast symptoms from diagnosis to 4 years later. Upset by hair loss had a relatively higher score at baseline, which increased significantly during follow-up, with a mean score of 42.95.

### Health care utilization

**Fig. 1:**
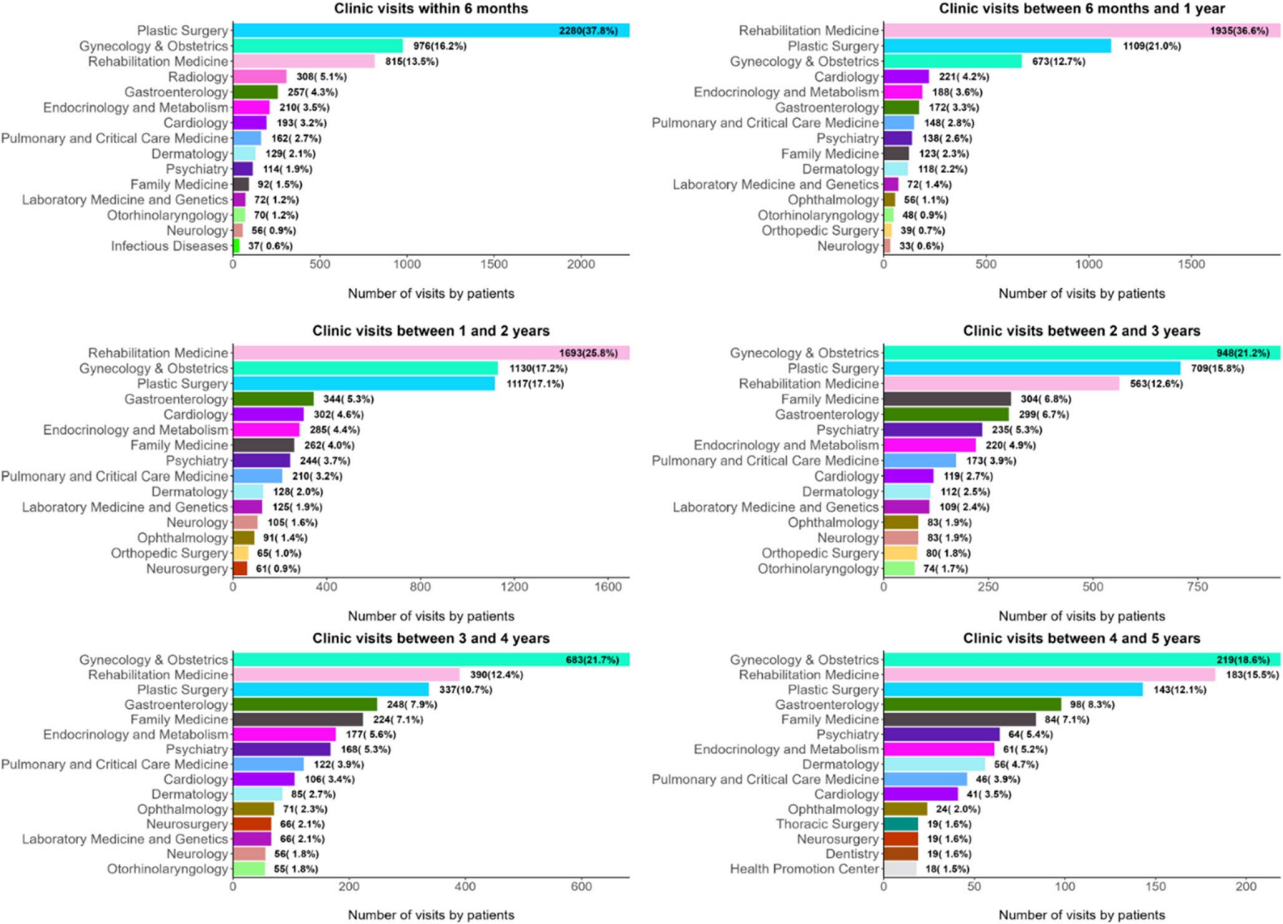
Clinical utilization over time since diagnosis

Besides a clinic visit for treatment, the most common department visited was plastic surgery (37.8%), followed by gynecology (16.2%). Within 6 months of diagnosis, the most frequent clinic was rehabilitation medicine (36.6%), followed by plastic surgery, gynecology, and cardiology. This trend was similar up to 2 years after diagnosis. After 2 years, the most common clinics were gynecology, family medicine, and psychiatry increased (Fig. 1).

## Discussion

This study describes a cohort profile of the BIG-S registry, a prospective cohort of 2,749 breast cancer survivors from Samsung Medical Center. The registry integrates detailed clinical data, patient-reported outcomes, and healthcare utilization patterns. Although emotional functioning was reduced, it improved. However, fatigue and insomnia persisted throughout the follow-up period, and cognitive and role functions decreased. Sexual function and breast-related symptoms, particularly distress over hair loss, have remained significant concerns over time. However, there were few clinic visits related to patient-reported outcomes, and the most frequent clinic visits were to gynecology and rehabilitation medicine.

While emotional functioning in relation to cancer is initially poor due to anxiety about cancer, it appears that once cancer treatment begins, this anxiety tends to decrease, leading to an improvement in emotional well-being [20]. This may be related to the initial uncertainty following a cancer diagnosis, which can cause significant anxiety [21], but as treatment plans are established and implemented, some of this uncertainty is resolved. Patients may understand their condition and the way forward, which may help reduce their anxiety [22]. However, emotional recovery is not universal. Survivors continue to experience emotional and psychological challenges [23], particularly related to fear of recurrence, fatigue, and difficulties in social reintegration [24]. The number of survivors who visited a psychiatric department increased several years after diagnosis. However, given that unmet emotional needs are higher at diagnosis, tailored psychosocial interventions and survivorship care planning could help address these issues and provide a more comprehensive approach to survivorship care.

From diagnosis to 4 years, fatigue and insomnia were prevalent problems in breast cancer survivors. A previous study on breast cancer survivors similarly identified fatigue and insomnia as prevalent and enduring problems, particularly among those with deteriorating quality-of-life trajectories​ [23]. Fatigue and insomnia may be affected by multifactorial factors that result from residual treatment effects, psychological distress, and underlying metabolic or hormonal changes induced by cancer therapies [25].

We observed that sexual function and upset hair loss posed significant challenges for survivors. In a previous cohort study, chemotherapy and endocrine therapy had substantial effects on body image, sexuality, and self-esteem​ [23]. These issues are particularly pronounced in younger survivors [23], for whom sexual dysfunction and hair loss may disrupt intimate relationships and hinder their return to normal. Despite the prevalence of these symptoms, limited engagement with related specialty clinics such as psychiatry and dermatology clinics highlights the gap in the current survivorship care model. This study noted that plastic surgery and rehabilitation medicine were commonly visited within the first 2 years after diagnosis, reflecting a focus on physical recovery and reconstruction. However, the increased visits to gynecology and psychiatry after 2 years suggest delayed recognition and management of psychosocial and sexual health needs. Therefore, early identification and interventions for symptom management are important [26]. However, they still lack access to specialized services that target persistent fatigue, insomnia, and sexual health. Delayed or insufficient access can exacerbate survivors’ struggles, particularly in systems lacking structured survivorship care pathways [27].

As survival rates for breast cancer continue to increase, long-term follow-up studies are essential to understand the persistent effects of treatment on female’s lives. This study provides insights into late-onset adverse effects such as chronic fatigue, cognitive changes, and psychological stress, all of which can hinder the process of returning to normal life. Furthermore, the contemporary nature of this cohort, which reflects the current multimodal treatment landscape, including new chemotherapeutic agents, endocrine therapies, and targeted treatments, enables a more relevant assessment of treatment burdens and their real-world implications for breast cancer survivors. By integrating socioeconomic data, this study sheds light on how financial and social factors interact with treatment outcomes, further influencing the abilities of females to reclaim their roles as mothers, wives, and individuals.

## Conclusions

Unlike previous breast cancer cohorts, the BIG-S comprehensively examined the evolving needs of breast cancer survivors from diagnosis to long-term survivorship. The study captures physical and psychological issues and social reintegration challenges, financial toxicity, and cancer adaptation. The BIG-S correlates self-reported patient experiences with actual healthcare utilization data, providing a holistic view of survivorship trajectories.

## Data Availability

The data supporting this study’s findings are available on request from the corresponding author (SKL). The data are not publicly available because they contain information that could compromise research participant consent.

## Abbreviations

EMR: Electronic medical records
EORTC QLQ-C30: European Organisation for Research and Treatment of Cancer Quality of Life Questionnaire-Core 30
ER: Estrogen receptor
FACIT: Functional Assessment of Chronic Illness Therapy
HER2: Human epidermal growth factor receptor 2
ICHOM: International Consortium for Health Outcomes Measurement
IHC: Immunohistochemical
PHQ9: Patient Health Questionnaire 9
PR: Progesterone receptor
PRO: Patient Reported Outcome
MRS: Menopause Rating Scale
SISH: Silver in situ hybridization
